# Epstein–Barr Virus and *Helicobacter Pylori* Co-Infection in Non-Malignant Gastroduodenal Disorders

**DOI:** 10.3390/pathogens9020104

**Published:** 2020-02-06

**Authors:** Ramsés Dávila-Collado, Oscar Jarquín-Durán, Le Thanh Dong, J. Luis Espinoza

**Affiliations:** 1 Faculty of Medicine, UNIDES University, Managua 11001, Nicaragua; 2 Faculty of Medical Technology, Hanoi Medical University, Hanoi 116001, Vietnam; 3 Faculty of Health Sciences, Kanazawa University, Kodatsuno 5-11-80, Kanazawa 920-0942, Ishikawa, Japan

**Keywords:** Epstein-Barr virus, gastritis, non-ulcerous peptic disease, peptic ulcer disease, *helicobacter pylori*

## Abstract

Epstein–Barr virus (EBV) and *Helicobacter pylori* (*H. pylori*) are two pathogens associated with the development of various human cancers. The coexistence of both microorganisms in gastric cancer specimens has been increasingly reported, suggesting that crosstalk of both pathogens may be implicated in the carcinogenesis process. Considering that chronic inflammation is an initial step in the development of several cancers, including gastric cancer, we conducted a systematic review to comprehensively evaluate publications in which EBV and *H. pylori* co-infection has been documented in patients with non-malignant gastroduodenal disorders (NMGDs), including gastritis, peptic ulcer disease (PUD), and dyspepsia. We searched the PubMed database up to August 2019, as well as publication references and, among the nine studies that met the inclusion criteria, we identified six studies assessing EBV infection directly in gastric tissues (total 949 patients) and three studies in which EBV infection status was determined by serological methods (total 662 patients). Due to the substantial methodological and clinical heterogeneity among studies identified, we could not conduct a meta-analysis. The overall prevalence of EBV + *H. pylori* co-infection in NMGDs was 34% (range 1.8% to 60%). A higher co-infection rate (EBV + *H. pylori*) was reported in studies in which EBV was documented by serological methods in comparison with studies in which EBV infection was directly assessed in gastric specimens. The majority of these studies were conducted in Latin-America and India, with most of them comparing NMGDs with gastric cancer, but there were no studies comparing the co-infection rate in NMGDs with that in asymptomatic individuals. In comparison with gastritis caused by only one of these pathogens, EBV + *H. pylori* co-infection was associated with increased severity of gastric inflammation. In conclusion, only relatively small studies testing EBV and *H. pylori* co-infection in NMGDs have been published to date and the variable report results are likely influenced by geographic factors and detection methods.

## 1. Introduction

Symptoms associated with upper gastrointestinal disorders such as epigastric pain and dyspepsia are common in general practice and represent a source of substantial morbidity, mortality, and health care cost [1]. Dyspepsia refers to a spectrum of symptoms that include recurrent pain or discomfort in the epigastric region, postprandial epigastric distension, and nausea or vomiting. These symptoms may be acute or chronic and account for a large number of doctor visits in primary care [2]. Etiologically, dyspepsia can be linked to various underlying disorders, such as gastroesophageal reflux disease (GERD), gastritis, peptic ulcer disease (PUD) or gallbladder diseases, however, for nearly one-third of cases, routine diagnostic procedures, including an upper endoscopy, do not identify an underlying organic or biochemical abnormality, and these cases are categorized as functional dyspepsia (FD) [2,3].

According to the recently revised Rome IV criteria, FD is defined by any combination of four symptoms (postprandial fullness, early satiety, epigastric pain, and epigastric burning) that interfere with usual individual activities, occurred at least 3 days per week in the last 3 months, and started at least 6 months prior to assessment, with no evidence of organic, systemic, metabolic or structural disease likely to explain those symptoms [4]. FD is one of the most prevalent functional gastrointestinal disorders (estimated worldwide prevalence of 5%–20%) and although it is not life-threatening, FD significantly impairs quality of life and increases health care expenses for both the patients and society [4]. The etiology of FD appears to be multifactorial, including *H. pylori* infection or gastrointestinal motility disorders that can be due to hypersensitivity to mechanical and chemical stimuli, immune activation, and elevated mucosal permeability in the proximal small intestine [4,5]. In addition, genetic predisposition and psychopathological comorbidity have also also proposed, although the evidence is weaker than has been observed in other functional gastrointestinal disorders, such as irritable bowel syndrome [5].

Gastritis is inflammation of the gastric mucosa that can be acute or chronic [1]. Acute gastritis involves areas of erosion of the mucosa of the stomach due to damage to mucosal defenses such as a reduction in protective prostaglandins caused by nonsteroidal anti-inflammatory drugs (NSAIDs), or may be the result of the effects of deleterious factors that directly include cellular necrosis. The two most important causes of gastritis are *H. pylori* infection and NSAIDs [6]. 

Chronic gastritis is the persistent and often progressive inflammation of the mucosa of the stomach that can be caused by either infectious (such as *H. pylori,* herpes virus, or cytomegalovirus) or noninfectious (such as NSAIDs, autoimmune gastritis, chemotherapy, or uremic gastropathy) conditions [7]. Prevalence of chronic gastritis has markedly declined in the developed world in recent decades but still remains an important cause of morbidity because long-term gastric inflammation can result in the development of peptic ulcer disease (PUD). Chronic gastritis can also lead to atrophic gastritis and gastric intestinal metaplasia (GIM), which are associated with mucosal dysfunctions and deficient absorption of essential vitamins, (vitamin B12) and micronutrients, such as iron, calcium, magnesium, and zinc. Both atrophic gastritis and GIM are considered precancerous conditions due to their strong association with gastric cancer [8]. 

PUD refers to an injury to the digestive tract caused by peptic acid, which results in a mucosal break (erosions or ulceration) reaching the submucosa. PUDs are usually located in the stomach or proximal duodenum, and less frequently in the esophagus [7,9]. About 10% of people develop PUD at some point in their life (annual incidence 0.1%–0.3%), however, the prevalence of this disorder, along with its associated hospitalization and mortality rates, are declining worldwide, particularly in developed countries, which has been attributed to the reduction in the burden of *H. pylori* infection and changes in the usage of NSAIDs [9]. 

Several studies have documented the simultaneous presence of EBV and *H*. *pylori* in gastric cancer specimens (ranging from 6% to 12%) and the significance of co-infection with both pathogens in gastric cancer was also assessed in meta-analysis [10,11,12,13]. Considering that persistent inflammation is associated with the development of cancer [14], one may speculate that individuals who develop gastric cancer in association with EBV or *H*. *pylori* may present with some degree of mucosal inflammation. Indeed, various studies have investigated the potential role of *H*. *pylori* and EBV co-infection in the development of gastritis and other non-malignant gastroduodenal disorders (NMGDs), including PUD, dyspepsia, and GERD, although variable results have been reported thus far [10,11,15]. We, therefore, conducted a systematic review to comprehensively evaluate publications in which EBV and *H. pylori* co-infections have been documented in patients with NMGDs. 

## 2. Biology and Disease Associations of *H. pylori* and EBV

*H. pylori* is a spiral-shaped gram-negative bacterium that has evolved various mechanisms to survive in the acidic environment of the human stomach. More than 50% of the global population has *H. pylori* in their upper gastrointestinal tract, with considerable geographical variations, with a higher prevalence in lower-income countries [16,17]. Infection with *H. pylori* is acquired (typically during early childhood) by the ingestion of contaminated food or water and is asymptomatic in up to 90% of people, however, some individuals develop acute gastritis, which may spontaneously resolve or may progress to chronic gastritis, PUD, or lead to premalignant lesions such as atrophic gastritis and GIM [18]. The lifetime risk of developing gastric cancer in *H. pylori-*infected individuals is 2% and thus this pathogen has been classified as Class-I carcinogen [19,20].

*H. pylori* express various factors that allow the bacterium to colonize and induce persistent infection in the stomach. For example, the adhesion membrane proteins, such as lipoproteins A and B (AlpA/B), blood group antigen binding adhesion (BabA), outer inflammatory protein A (OipA) and sialic acid-binding adhesion (SabA), allow the bacterium to adhere to the gastric epithelium via receptor-mediated adhesion mechanisms [21]. The presence of flagella and the enzyme urease, which hydrolyzes urea releasing ammonia, thus neutralizing the acidic gastric environment, also contribute to *H. pylori* colonization of the stomach. In addition, *H. pylori* express various virulence factors that can induce cellular damage predominantly through the effects of secretory toxins, including vacuolating cytotoxin A (VacA) and cytotoxin-associated gene A (CagA) [22]. 

EBV is a γ-herpes virus that infects nearly 90% of adults around the world and is etiologically associated with several lymphoid malignancies, including Hodgkin lymphoma, Burkitt’s lymphoma, a subset of diffuse large B-cell lymphomas, and nasal T/NK cell lymphomas [23,24]. The virus, which is most often acquired during infancy by contact with the saliva of infected individuals, firstly infects epithelial cells of the nasopharynx and thereafter spreads to B lymphocytes, and ultimately establishes lifelong latent infection in memory B cells. Acute EBV infection is usually asymptomatic or may cause a febrile benign infection, however, in some individuals, especially when first infected during adolescence, the virus causes the typical infectious mononucleosis syndrome with fever, malaise, and lymphadenopathy. EBV latency is categorized into three types: latency I, latency II, and latency III, based on the array of viral genes that are expressed. The host immune response, mostly mediated by cytotoxic T lymphocytes (CTL), plays an essential role in preventing infected cells from switching to a lytic stage with productive viral replication. This is illustrated by the frequent reactivation of EBV infections observed in immunodeficiency conditions such as post-transplant lymphoproliferative disease, in which EBV-infected B-cells proliferate due to therapeutic immunosuppression after organ transplantation [25,26]. 

EBV can also infect epithelial cells, including cells of the gastric mucosa, where it may induce acute or chronic gastritis [11], and accumulating evidence appears to link the virus with the development of gastric cancer [27]. 

An increased number of reports suggest that some sort of cooperation exists between EBV and *H. pylori*, where the presence of one of these microorganisms may promote the growth of the other and vice versa, and could also increase their virulence. Although the mechanisms controlling this synergistic interaction are not entirely known, several lines of evidence suggest that during the course of co-infection with *H. pylori* and EBV, immune cell recruitment to the site of infection is considerably increased, which potentiates gastric inflammation and tissue damage. For example, monochloramine, an oxidant produced in the stomach in the presence of *H. pylori* infection, can induce the conversion of EBV from the latent to the lytic phase [28]. On the other hand, pro-inflammatory cytokines arising in the course of gastric inflammation induced by *H. pylori* may contribute to the proliferation of EBV. For example, interferon γ (IFN-γ) secretion induced by *H. pylori* promotes an inflammatory milieu that exacerbates disease severity [29], and this cytokine, along with IL-6 and IL-13, promote EBV proliferation, and higher levels of proinflammatory cytokines, including IL-1β, tumor necrosis factor α (TNF-α) and IL-8, have been reported to promote severe gastritis associated EBV and *H. pylori* co-infection [10]. In line with these observations, IFN-γ levels in the plasma of patients with gastric cancers are positively correlated with the degree of EBV reactivation [30]. 

Furthermore, persistent activation of Th17 cells appears to be implicated with the gastric inflammation associated with *H. pylori* and EBV co-infection. Th17 cells are a subset of helper CD4+ T cells with proinflammatory properties that activate innate immune cells, regulate B cell responses, and participate in antimicrobial immune responses and wound healing. Th17 cells and their key cytokine, IL-17A, are implicated in the pathogenesis of gastritis induced by *H. pylori* [31]. For example, animal studies have shown that EBV directly induces the secretion of the proinflammatory cytokine IL17 [32]) and higher neutrophil infiltration within the submucosa and lamina propria of the stomach in *H. pylori-*infected wild type compared to their *H. pylori*-infected IL-17A−/− counterparts [33], thus suggesting that IL-17A is required for neutrophil infiltration. Similarly, increased levels of IL-17A and IL-8 were observed in the gastric mucosa of gastric ulcer and non-ulcer *H. pylori*-infected patients compared to uninfected non-ulcer patients, and both IL-17A and IL-8 are strongly correlated with an increase in neutrophil infiltration in infected patients. Interestingly, serum levels of IL-17 directly correlate with the EBV DNA load in individuals with rheumatoid arthritis, which was not observed in controls, and this IL-17 enhancement, in the course of EBV infection, is mediated by toll-like receptor 9 (TLR9) [34]. 

IL-21 is a pleiotropic proinflammatory cytokine secreted by Th17 cells, as well as by T follicular helper cells and NK cells or Th1 cells. IL-21-deficient mice showed impaired immune cell infiltration in the gastric mucosa in response to *H. pylori* [35] and human studies have shown that IL-21 is produced in the gastric mucosa in response to *H. pylori* infection and its expression level appears to correlate with the severity of gastric inflammation [36,37]. Interestingly, IL-21 was able to directly activate gastric epithelial cells leading to the upregulation of matrix metalloprotease 1 (MMP-1) and MMP-3 on the gastric cell line AGS cells and in fibroblast-like synoviocytes [36,38], which may contribute to tissue damage. Notably, IL-21 was expressed in circulating CD4+ and CD8+ T cells in a patient with lymphoproliferative disorder associated with EBV infection, which was associated with severe tissue damage [39]. 

## 3. Material and Methods

This study was conducted following the PRISMA (Preferred Reporting Items for Systematic Reviews and Meta-Analyses) statement [40]. We used PubMed database to search articles listed on or before September 30, 2019, using the following search terms “Epstein Barr Virus, *helicobacter pylori* and gastritis”, “EBV, *helicobacter pylori* and gastritis,” “Epstein Barr Virus, *helicobacter pylori* and duodenitis”, “EBV, *helicobacter pylori* and duodenitis”, “Epstein Barr Virus, *helicobacter pylori* and gastrointestinal inflammation”, “EBV, *helicobacter pylori* and gastrointestinal inflammation”, “Epstein Barr Virus, *helicobacter pylori* and non-ulcer peptic disease”, “EBV, *helicobacter pylori* and non-ulcer peptic disease”, “Epstein Barr Virus, *helicobacter pylori* and peptic ulcer disease”, “EBV, *helicobacter pylori* and peptic ulcer disease,” “Epstein Barr Virus and *helicobacter pylori* coinfection”, “EBV and *helicobacter pylori* coinfection”, “Epstein Barr Virus, *helicobacter pylori* coinfection and gastrointestinal disorders” “EBV, *helicobacter pylori* coinfection and gastrointestinal disorders”, “Epstein Barr Virus, *helicobacter pylori* coinfection and gastroduodenal inflammation” “EBV, *helicobacter pylori* coinfection and gastroduodenal inflammation”, “Epstein Barr Virus, *helicobacter pylori* coinfection and gastritis” “EBV, *helicobacter pylori* coinfection and gastritis”. In addition, references listed in eligible articles were further scrutinized for identifying studies missed in the primary screening. 

### 3.1. Study Selection and Data Extraction 

**Figure 1:**
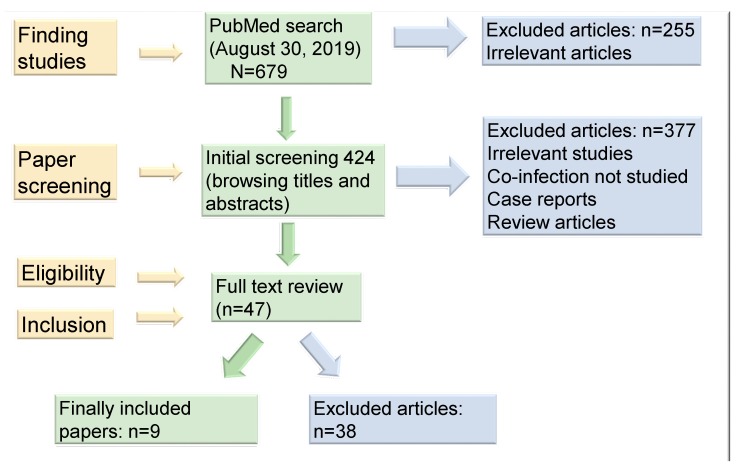
Outline of the article search and study selection.

A flow diagram of the study selection process is shown in Figure 1. Prospective studies were selected by three independent researchers (JLE, RDC, and OJD). Titles and abstracts were browsed during a primary selection and potentially eligible studies were subject to full-text review. Discrepancies regarding study inclusion or exclusion were resolved by further review and discussion. Eligibility criteria for inclusion were (1) the search was limited to studies in humans; (2) studies must have ascertained EBV status of gastroduodenal tissue using (EBV-encoded RNA) EBER in situ hybridization, PCR, or serological methods that detect EBV infection; and (3) studies that reported *H. pylori* positivity in gastroduodenal specimens. Extracted items included general study characteristics (year, country, study design), characteristics of the study populations (size, sex, age, disease-related factors), and types of measurements (specimen types, analytic procedures). The number of cases was extracted from all publications or, in some cases, calculated from the reported percentage of cases. 

### 3.2. Studies Included 

A total of 679 titles were identified in the first round of data retrieval and 255 of them were rapidly excluded because their titles were clearly irrelevant, even though they had the keywords “EBV and *Helicobacter pylori* co-infection”. After browsing titles and abstracts, 377 articles were further excluded because some were case reports, others were conference proceedings, some were review articles, some included only gastric cancer patients and, in some studies, co-infection was not studied. The remaining 47 articles were subjected to full-text review and finally, nine papers met the inclusion criteria and were then systematically reviewed. Due to the excessive clinical diversity and substantial methodological heterogeneity among studies, as well as the small number of patients enrolled in most studies, with low statistical power, we could not perform a meta-analysis. 

## 4. Results 

After extensive review, we identified nine studies that met the selection criteria. The flow chart of the literature searches and study selection strategy is shown in Figure 1. Among the selected studies, EBV was directly detected in gastric tissues in six studies and, in three studies, serological methods that detect EBV infection were utilized. Notably, except for a study conducted in Hungary, all studies were conducted either in Latin America (Mexico, Peru, Paraguay, and Brazil) or in India (three studies). In total, 1611 individuals with NMGD were included in the present systematic review. Among them, 662 were from studies assessing active EBV infection by serological methods and 949 were from studies in which EBV infection was directly documented in gastric tissues. 

### 4.1. Studies Assessing EBV by Serological Methods.

Serological diagnosis of EBV infection is based on the utilization of specific antibodies that recognize EBV antigens as determining disease status, however, given the ubiquitous distribution of the virus, infecting nearly 95% of adult worldwide population, and the high degree of interindividual variability, studies assessing EBV infection by serological methods must be interpreted cautiously. Three viral antigens, the viral capsid antigens (VCAs), the early antigens (EAs), and the Epstein–Barr nuclear antigens (EBNAs), are typically detected in serological studies. In general, EBV infection status is determined by testing more than one parameter, which provides reasonable criteria to distinguish an acute from a past EBV infection. For example, whereas the presence of anti-VCA IgG and IgM antibodies indicates active acute infection if anti-EBNA 1 is not detected [41], the presence of anti-VCA IgG and anti-EBNA IgG positivity in the absence of anti-VCA IgM antibodies suggests a past (non-active) infection [42]. The serological results and interpretation are listed in Table 1. 

We identified three studies in which EBV infection status was assessed by serological methods (Table 2). A prospective study conducted in Hungary assessed the prevalence of *H. pylori* and EBV co-infection in 104 patients aged between 18 and 80 years with benign upper digestive diseases, as confirmed by endoscopy [43]. EBV infection status was assessed by serology and *H. pylori* infection was determined by the modified Giemsa stain and by IgG-chemiluminescence. Co-infection with *H. pylori* and EBV was significantly more prevalent in patients with duodenal ulcer (60%), while in functional dyspepsia (18.1%) and reflux (12.9%), infection prevalence was similar to that of the general population. The major limitations of this study include the low number of cases enrolled (total 104), with less than 50 cases in each subgroup (PUD 40, FD 33 and 31 RD), which considerably impairs the statistical power of the analysis. In addition, the fact that infection status was assessed by serological methods may have contributed to overestimating the co-infection rate. This is especially relevant considering the fact that EBV was diagnosed based only on IgG and IgM against VCA, and IgG anti-EBNA1 were not included in the analysis, which makes it difficult to distinguish between active infection and past infection. This may suggest that some cases categorized as EBV-seropositive did not have an active EBV infection. This notion may be supported by the fact that, according to “laboratory database” cited by the authors, the overall seroprevalence of *H. pylori* in the general population of the district where the study was conducted (the year 2014) was 29.4% (2385 out of 8107 cases), while EBV IgG was positive in 62.9% (2254 out of 3578 cases) and VCA IgG positivity was 79.4% (2825 out of 3556 cases), and seroprevalence for both pathogens in this population was 27.9% (59 out of 211 cases). Interestingly, a study by the same group reported a gradual decrease in the prevalence of *H. pylori* infection from 71.3% to 32.7% between 1997 and 2012 [44].

A study conducted in Mexico showed that co-infection with EBV and *H. pylori* in children is associated with severe gastritis [10]. The study included 333 pediatric patients (0–17 years old) with recurring abdominal pain and the frequency of EBV infection, assessed by IgG and IgM antibodies against VCA, was 64.3% and *H. pylori* infection determined by IgG antibodies against *H. pylori* whole-cell extracts was 53.4%. Importantly, children infected only by EBV presented mild mononuclear cell (MNC) and polymorphonuclear cell (PMNC) infiltration, while those infected by *H. pylori* presented moderate MN and mild PMN. In contrast, patients co-infected with both pathogens were significantly associated with severe gastritis. Of note, 33.9% of patients presented antibodies against CagA, however, the sole infection with *H. pylori* CagA+ strains was not sufficient to cause a severe inflammatory response in the absence of EBV infection. These observations suggest that, at least in the pediatric population, *H. pylori* infection alone may not be sufficient to cause severe gastritis and point to a synergistic or cooperative effect of EBV and *H. pylori* in the pathogenesis of gastric inflammation. 

The same group conducted a case-control study, in which antibodies against EBV, *H. pylori* and CagA were analyzed for association with the type of gastric lesion and the degree of inflammation [45]. This study included 525 adult patients (≥30 years old) from two Latin American countries (309 from Mexico and 216 from Paraguay). Among them, 225 samples (42.9%) were categorized as NAG, 186 samples (35.4%) were classified as premalignant lesions and 114 (21.7%) as gastric cancer. Importantly, seropositivity for both pathogens (*H. pylori*+/EBV+) in this study was very high (434/525); while 63 were seropositive only for EBV, 25 were positive for *H. pylori*, and three patients were double negative. A significantly increased risk was observed for premalignant lesions when *H. Pylori*+/EBV+ was compared with *H. pylori*+ alone (OR = 8.4, 95% CI 1.8–38.9), as well as with EBV infection alone (OR = 2.0; 95% CI 1.03–3.9). Importantly, EBV + *H. pylori* coinfection was also significantly associated with increased mononuclear cells and polymorphonuclear cells’ (PMNC) infiltration in gastric tissues compared with single infection. It must be noted that since this study was based on serological methods, the lack of microbiological or molecular evidence supporting the presence of these pathogens in gastric tissues is a major limitation. In addition, the fact that EBV infection status was solely based on one serological parameter (IgG anti-VCA) may account for the elevated co-infection rate reported in the studied population. In line with this observation, whereas most patients were positive for IgG antibodies, only 120 (22.9%) were also positive for IgA anti-VCA.

As mentioned above, EBV infection status is determined by testing more than one parameter to accurately distinguish acute from a past EBV infection, thus, considering that IgM anti-VCA measurements were not included in this study, as the authors stated, “because only a few samples were positive, and IgM-positive patients preferentially presented NAG, we did not continue that analysis”, based on the available data, the proportion of patients with an active EBV infection is unknown. In addition, the study included serum samples from 129 asymptomatic individuals (median age = 41.6 ± 7.7; 0.98 male to female ratio) that were tested for anti-VCA and anti-*H. pylori* antibodies titers. In this group, the median antibody titers were 72.7 for anti-VCA IgG, 1.2 for anti-*H. pylori* IgG, and 1.0 for anti-CagA IgG. Unfortunately, further analysis in this group was not pursued. For example, data on the seroprevalence of antibodies against both pathogens in this group were not shown.

### 4.2. Studies EBV Infection Directly Assessing in Gastric Tissues

The presence of EBV and *H. pylori* in gastric tissues was assessed in six studies (Table 3). A study conducted in India prospectively studied the association of *H. pylori* and EBV in patients with gastric cancer and PUD. For that purpose, 348 adult patients (non-ulcer dyspepsia (NUD) 241, PUD 45, GC 62) undergoing upper gastrointestinal endoscopy between September 2003 and May 2007 were enrolled in the study [46]. Interestingly, *H. pylori* infection rate, diagnosed by rapid urease test, culture, histopathology, and PCR, was significantly higher in patients with PUD than in those with gastric cancer (80% versus 56.5%, *p* = 0.01) and NUD (80% versus 55.2%, *p* = 0.002). On the other hand, EBV DNA, which was detected by PCR for EBNA-1 gene and sequence analysis, was more frequently detected in patients with gastric cancer and PUD than in those with NUD. Coinfection with both pathogens was more frequent in patients with PUD (62.2%) in comparison with gastric cancer (46.8%) and NUD (29.5%). An intriguing finding of this study was the high prevalence of EBV DNA (50.3%) in the study population, which is not consistent with that reported in other studies, where the detection rate of EBV DNA in patients with gastric cancer ranges from 4.6% to 16% [45,47,48]. Potential technical issues associated with the PCR method utilized to detect the EBV DNA may have contributed to such a high virus detection rate, however, in this study, a fraction of the samples that tested positive for EBNA-1 were also confirmed by sequence analysis, which appears to support the veracity of the findings. 

In another study from India, biopsy samples were collected from 200 adult patients undergoing upper gastrointestinal endoscopy and diagnosed as NUD (100 cases), PUD (50), and gastric carcinoma (50) [52]. Infection rate with *H. pylori* in gastric specimens, diagnosed by rapid urease test, culture, histopathology, PCR and quantitative PCR, was significantly higher in patients with PUD than in those with GC (*p* = 0.044) and NUD (*p* < 0.001); however, no difference was observed between gastric cancer and NUD (*p* = 0.083). The frequency of EBV DNA (detected by PCR for EBNA-1 gene) in the study population was 56.5%, and the overall co-infection rate in the studied population was 40.5%. This was significantly higher in patients with gastric cancer and PUD than in those with NUD (*p* < 0.001). 

Importantly, the median EBV copy number in *H. pylori-*infected patients was significantly higher than in uninfected patients, however, no difference was observed between *H. pylori-*infected versus non-infected patients when data were analyzed according to disease subsets. 

The increased EBV DNA load in *H. pylori*-infected patients may support the notion that *H. pylori* promotes the reactivation of EBV. This hypothesis was tested in another study by the same group, which included 200 adult patients undergoing upper gastrointestinal endoscopy [51]. The prevalence of EBV infection was higher in patients with gastric cancer and PUD than in those with dyspepsia and whereas levels of EBNA1 transcripts, detected in all EBV positive cases, were not associated with disease type, the expression of BZLF1 was significantly associated with gastric cancer and PUD compared to dyspepsia (*p* < 0.01). Interestingly, BZLF1 expression was significantly higher in *H. pylori-*infected patients and other viral lytic factors, BARF1 and BcLF1, were significantly higher in the gastric epithelium of patients with severe chronic inflammation and gastric atrophy, respectively. Thus, the increased expression of lytic transcripts in patients with gastric cancer and *H. pylori* infection suggests the association of this bacterium with EBV reactivation and points to the pathogenic role of EBV reactivation in the pathogenesis of the diseases. 

A retrospective study conducted in Brazil investigated the presence of *H. pylori* (detected by urease test and PCR for CagA) and EBV (detected by Eber-1 in situ hybridization) in gastric specimens from 226 individuals randomly collected during the period 2005–2013 [50]. The study included 62 juvenile patients (12 months to 18 years old) and 39 adults (19 to 61 years old), referred for endoscopic examination to clarify upper gastrointestinal symptoms, as well as tumor samples from 125 adults (26 to 89 years old) with primary gastric adenocarcinoma. Among the juvenile patients, 53 (85.5%) showed some degree of gastritis (45.3% with mild gastritis and 54.7% with moderate/severe gastritis), while all the adult individuals presented gastritis. *H. pylori* infection was detected in 58.5% of samples from juvenile patients, 69.2% of adult gastritis samples and 88% of patients with gastric cancer and was not detectable in normal gastric mucosa. This study found no association between EBV and *H. pylori* co-infection with any clinicopathological variable, which may be due to the low EBV detection rate, as the virus was detected in only 3.8% of samples from juvenile gastritis patients, 5.1% of samples from adult gastritis patients and 9.6% of gastric patients. 

A study conducted in Mexico assessed the presence of EBV, human cytomegalovirus (HCMV) and *H. pylori*, all detected by PCR methods, in gastric specimens from patients with chronic gastritis (106 cases) and gastric cancer (32 cases) [15]. Overall, 73.9% of gastric biopsies were positive for EBV, while 52.9% and 46.4% were positive for HCMV and *H. pylori,* respectively. Interestingly, whereas 53% of *H. pylori*+ patients with chronic gastritis were also positive for EBV and 33% were both EBV+/HCMV+, the majority (92.3%) of patients with gastric cancer and *H. pylori* infection were EBV+ and 46.1% were EVB+/HCMV+. The authors proposed that these pathogens may act synergistically to induce inflammation and gastric cancer, although no direct evidence, such as histological studies and multivariate analysis, was provided to support such an assumption [15]. 

Although isolated case reports linking CMV with gastric inflammation have been reported, including in cases of documented co-infection with *H. pylori* [53,54,55], this was the first study to report such a relatively high frequency of both pathogens in gastritis and gastric cancer. Important limitations in this study were the small size of the studied population, a highly heterogeneous population (age range 4–89 years old), as well as the lack of a healthy control group and the fact that all pathogens were detected by PCR methods. It is worth mentioning that prevalence of EBV in individuals with chronic gastritis in the study population (69.8%) was significantly higher than reported in previous studies conducted in other regions of the country, which were about 10% [11], although the authors did not discuss the potential cause of that discrepancy.

Recently, a study conducted in Peru aimed to determine the prevalence of *H. pylori* and EBV and its association with the clinicopathological features of gastric cancer and chronic gastritis [49]. This single-center study included 540 patients (median age was 60 years) evaluated between 2015 and 2018, with 375 of them having gastric cancer (67 underwent diagnostic endoscopy and 308 gastrectomy) and 165 chronic gastritis cases who underwent diagnostic endoscopy. Gastric specimens were collected from old patients for further analysis. Prevalence of *H. pylori*, assessed by the detection of *hspA* and *UreA* genes via PCR, was 62.9% in the whole population and 60.8% in the gastric cancer subset. The prevalence of EBV, assessed by PCR for the detection of the BNRF1 gene, in gastric tissues of the whole population was 14.1%, and was higher in the gastric cancer group compared with the chronic gastritis subset (19.2% vs. 2.4%; *p* < 0.001) This EBV infection rate is substantially higher than reported (3.9%) in a previous study in the same population, in which the virus was detected by in situ hybridization [56], but is similar to the average infection rate reported in patients with gastric cancer in South American countries (10%). Coinfection of both pathogens was found in 42 patients (7.8%) in the whole studied population, being significantly higher in gastric cancer compared with chronic gastritis (10.7% vs. 1.2%; *p* < 0.001). The frequency of *H. pylori* strains expressing the virulent factor CagA in this study was lower (79.9%) than reported 10 years ago in the same population [57], and lower than reported in Brazil (96.7%) [58]. 

Limitations associated with this study include the fact that both pathogens were detected by a single detection method (PCR); the lack of baseline data, namely infection rates in the healthy population; and the use of convenience sampling, instead of paired selection, since subjects included in the study were patients with gastric cancer and chronic gastritis who were evaluated in a centralized institute for diagnosis.

## 5. Discussion

Although a few meta-analyses assessing the association of either *H. pylori* or EBV with gastric cancer have been published to date, and even a systematic review which revised articles reporting on co-infection rate with both pathogens in patients’ gastric cancer [12,27,59], to the best of our knowledge, this is the first study to systematically review the literature testing the association between *H. pylori* or EBV co-infection with NMGDs. We identified only nine clinical and epidemiological studies that complied with our inclusion criteria and among them, three studies assessed EBV infection rate by detecting anti-EBV antibodies in blood samples and six studies detected EBV positivity directly in gastric tissues, mostly by PCR methods. 

We observed a high variability in the prevalence of EBV and *H. pylori* in patients with NMGDs among the studies included in this review, that is directly related to the detection methods utilized to determine infection status. As expected, an increased EBV infection rate in NMGDs was observed when the virus was detected by serological markers in blood samples, and, although these methods may be useful to evaluate cumulative lifetime exposure and reactivation of the viral infection, when seropositivity is assessed only by detecting EBV antibodies that do not distinguish active infection from life-time infection (usually acquired from childhood), determining EBV infection status is challenging. In this case, comparing EBV antibodies titers between NMGDs patients with those in asymptomatic controls might provide more reliable data to judge the association of EBV infection with NMGDs. Similarly, increased EBV positivity was also reported in studies in which the virus was detected in gastric tissues by PCR methods. It is well-known that, whereas PCR methods are more sensitive than the gold-standard ISH method to detect EBV, they are also associated with a lower specificity. In addition, the PCR method is not able to distinguish EBV DNA present in epithelial cells from that found in infected lymphocytes infiltrating the affected tissue specimens. Therefore, since a potential EBV cross-contamination picked up by PCR methods cannot be completely ruled out, the increased EBNA-1 positivity detected by PCR, particularly in chronic gastritis specimens, rather than reflecting epithelial cells’ infection, could indicate the severity of an inflammatory response. 

Is there any specific clinical picture associated with EBV and *H. pylori* co-infection? It is unlikely that simultaneous infection with both pathogens causes a defined clinical entity, however, some studies suggest that that co-infection with EBV and *H. pylori* induces more severe inflammatory responses in individuals with gastritis, which ultimately increases the risk of developing the intestinal-type gastric cancer [45]. Mechanistically, both pathogens promote immune cell influx and the secretion of pro-inflammatory cytokines in the gastric mucosa. For example, *H. pylori*, mainly via the virulence factor CagA, promotes cell proliferation and reduces apoptosis, supporting the establishment of a chronic inflammatory milieu via the activation of multiple host cell signal pathways such as ERK/MAPK, NF-κB, P13K/Akt, and JAK/STAT3 pathways [60,61,62] and, as mentioned above, NH2C produced by *H. pylori* promotes the transition of EBV from the latent to the lytic phase, which promotes virus reactivation [28]. 

Both *H. pylori*- and EBV are capable of inducing epigenetic changes in target cells, leading to oncogene activation or tumor suppressor gene silencing, which increases the risk of malignant transformation [63,64,65]. For example, Src homology region 2-containing protein tyrosine phosphatase 2 (Shp2), encoded by PTPN11, plays an important role in signal transduction downstream of growth factor receptor signaling, and is associated with various cancer types, including gastric cancer. Interestingly, tissue specimens of EBV+ gastric cancers exhibited SHP1 hypermethylation with reduced SHP1 expression, and the infection of gastric epithelial cells with EBV induces SHP1 promoter hypermethylation, which strengthens phosphorylation-dependent CagA action [66]. 

**Figure 2:**
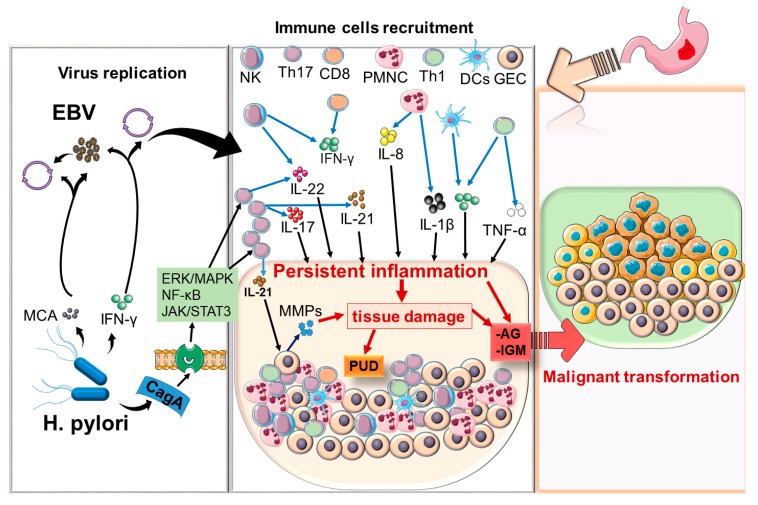
Interaction between EBV and *H. pylori* and gastric inflammation and its potential association with gastric cancer.

Based on the above considerations, there is a synergistic relationship between EBV and *H. pylori* which potentiates the local inflammatory response, ultimately leading to increased tissue damage (Figure 2). 

*H. pylori*-associated monochloramine (MCA) induces EBV lytic conversion in gastric epithelium harboring EBV latent infection. Active EBV infection induces an inflammatory response in the gastric mucosa by attracting immune cells, including Polymorphonuclear cells (PMNCs), natural killer cells (NK cells), and dendritic cells (DCs), as well as CD8, Th17 and Th1 cells. The inflammatory response is further potentiated by *H. pylori*, which expresses several virulent and proinflammatory factors. For example, CagA induces the activation of various signals pathways, including NFκB, MAPKs, and JAK/STAT3, which also contribute to the generation of a proinflammatory milieu that includes several components of the innate immune system (PMNCs, NK cells, etc.), along with infiltrating lymphocytes, especially Th1, Th17, Th22, and their associated proinflammatory cytokines (IL-1β, TNFα, IFNγ, IL-17, IL-21, IL-22) leading to severe tissue damage. In addition, IL-21 can directly activate gastric epithelial cells (GECs), which secrete matrix metalloproteases (MMPs) that further cause tissue damage. The establishment of a persistent or chronic infection can lead to the development of peptic ulcer disease (PUD) or premalignant lesions such as atrophic gastritis (AG) or gastric intestinal metaplasia (GIM), which eventually result in the development of gastric cancer. 

A major limitation of the published studies is that they were mainly focused on determining a potential correlation between EBV and *H. pylori* co-infection with the development of gastric cancer, and thus patients with benign disorders were included as controls. We found no studies comparing the prevalence of EBV and *H. pylori* co-infection in NMGDs with that in healthy subjects. This is in part due to the technical and ethical issues associated with including asymptomatic individuals as a control group to determine the precise co-infection rate in healthy individuals, because obtaining gastric specimens requires the use of invasive methods (endoscopy), which are not routinely performed in this population. Considering that both *H. pylori* and EBV are ubiquitous microorganisms infecting a broad proportion of the world population, and although various studies have suggested that a potential association exists between co-infection with these agents and the development of a more severe gastric mucosal damage, it is somewhat surprising to find that, with the exception of a small study from Hungary, all studies investigating this issue have been conducted in developing countries, including three studies from India, and five from Latin America. Moreover, gastric carcinogenesis is a multistep process that very often, if not always, initiates with chronic inflammation, which not only promotes tumor initiation but also plays a critical role in tumor growth and metastasis. Therefore, elucidating the mechanisms implicated in the development of acute or chronic infection in the context of a co-infection with EBV and *H. pylori* is crucial for designing cancer prevention strategies. Furthermore, the potential association of both pathogens in the development of NMGDs should also be investigated in other populations, including Caucasians, Asians, and Africans.

Clinical and epidemiological evidence supports the contribution of various viruses, including EBV, hepatitis virus, cytomegalovirus with gastritis and gastric cancer induced by *H. pylori* [67], and several studies have shown that gastric microbiota can regulate *H. pylori* infection and vice versa; this bacterium can modify the gastric microbiota composition [68,69,70]. On the other hand, the microbiota composition in several body niches exerts direct or indirect effects on virus infections, including EBV and human papilloma virus [71]. Therefore, testing potential crosstalk between EBV and *H. pylori* with gastric microbiota is an interesting line of investigation for further studies.

## Figures and Tables

**Table 1:** Serological parameters and interpretation for assessing Epstein–Barr Virus (EBV) infection status [41,42].

Antibody	Active Infection	Past Infection	Reactivation
VCA IgM	positive	negative	negative
VCA IgG	positive	positive	positive
EBNA IgM	positive	negative	positive
EBNA IgG	negative	positive	positive
EA-D IgG	negative	negative	positive

**Table 2:** Prevalence of EBV and HP co-infection (serological studies).

Study/Country	Method	Disease	No. Tested	EBV Positivity	*H. pylori* Positivity	Co-Infection Positivity	Key Findings
Buza´s et al. 2015 [43] Hungary	EBV: ELISA test (IgG and IgM) against EBV viral capsid protein (VCA) *H. pylori*: -Giemsa stain -IgG-chemiluminescence	PUD	40	75%	72.5%	60%	Higher prevalence of H. *pylori* + EBV co-infection along with higher anti-IgG levels found in duodenal ulcer. Could be attributed to an increased viral load or a stronger immune response
		FD	33	51.2%	33.3%	18.1%	
		GERD	31	51.6%	25.8%	12.9%	
		Overall	104	70.1%	56.7%	30%	
Cárdenas-Mondragón et al. 2012 [10]. Mexico	EBV: ELISA test (IgG and IgM) against EBV VCA *H. pylori*: ELISA test (IgG) against *H. pylori* whole-cell extracts and against CagA protein	Non-atrophic gastritis (NAG)	333 pediatric patients (median age 10.1 ± 3.7)	64.3%	53.4%	1.8 (1–11.8)	EBV + *H. pylori* co-infection: significantly associated with severe gastritis. 2.4% (1%–9.7%)
Cárdenas-Mondragón et al. 2015 [45] Mexico and Paraguay	EBV: ELISA test (IgG and IgM) against EBV VCA *H. pylori*: ELISA test (IgG) against *H. pylori* whole-cell extracts and against CagA protein	Non-atrophic gastritis (NAG)	225 patients (median age 30)	32 (14.3%)	18 (8%)	175 (77.7%)	EBV collaborates with *H. pylori* to induce severe inflammation, increasing the risk of malignant transformation

**Table 3:** Prevalence of EBV and *H. pylori* co-infection (microbe detection in gastric tissues).

Reference/Country	Tissue/Method	Disease	No. Tested	EBV (%) Positivity	*H. pylori* Positivity	Co-Infection Positivity	Caga Positivity
[49] Castaneda et.al. Peru	Gastric biopsy specimens *H. pylori*: qPCR detection of hspA and UreA genes EBV: PCR detection of BNRF1 gene	Chronic gastritis	165	4 (2.4)	112 (67.9)	2 (1.2)	
		Comparative samples: GC	375	72 (19.2)	228 (60.8)	40 (10.7)	
		Overall	540	76 (14.1)	340 (63.0)	42 (7.8)	
[15] Moral-Hernández et al. 2019. Mexico.	Gastric biopsy specimens *H. pylori*: 16S rRNA gene detection by PCR EBV: PCR detection of EBNA1 gene	Chronic gastritis	106	74 (69.8)	51 (48.1)	27 (25.4)	
		Comparative samples: gastric cancer	32	30 (87.5)	13 (40.6)	12 (37.5)	
		Overall	138	104 (75.4)	64 (46.4)	39 (28.2)	
[50] de Souza et al. 2014. Brazil	Gastric biopsy specimens *H. pylori*: Rapid urease test and PCR EBV: Eber1 detection by ISH	Juvenile patients with upper gastrointestinal symptoms	62	2 (3.2)	31 (50)	1 (1.6)	20 (32.3)
		Comparative samples: adults with similar symptoms	39	2 (5.1)	27 (69.2)	2 (5.1)	20 (51.3)
		Comparative samples 2: adults with gastric cancer	125	12 (9.6)	110 (88)	12 (9.6)	84 (67.2)
[51] Shukla et al. 2012. India	Gastric biopsy specimens *H. pylori:* Rapid urease test and PCR for urea gene EBV: PCR for detection of EBNA-1 gene and also BZLF1, BARF1 and BcLF1 genes	Non-ulcer dyspepsia	120	36 (30)	Unreported	Unreported	Unreported
		PUD	30	19 (63.3)	Unreported	Unreported	Unreported
		Comparative samples: gastric cancer	50	40 (80)	Unreported	Unreported	Unreported
		Overall	200	95 (47.5)	105 (52.5)	56 (28)	Unreported
[52] Shukla et al. 2011. India	Gastric biopsy specimens *H. pylori:* Rapid urease test and PCR for urea gene EBV: PCR for detection of EBNA-1 gene	Non-ulcer dyspepsia	100	37 (37)	46 (46)	23 (23)	
		PUD	50	35 (70)	41 (82)	31 (62)	
		Comparative samples: gastric cancer	50	45 (90)	31	27	
		Overall	200	117 (58.5)	118 (59)	81 (40/5)	
[46] Saxena et al. 2008. India	Gastric biopsy specimens *H. pylori*: Rapid urease test and PCR for urea gene EBV: PCR for detection of EBNA-1 gene *H. pylori* Ureasa test PCR	Non ulcer dyspepsia	241	90 (37.3)	133 (55.2)	71 (29.5)	
		PUD	45	34 (75.6)	36 (80)	28 (62.2)	
		Comparative samples: gastric cancer	62	51 (82.3)	35 (56.5)	29 (46.8)	
		Overall	348	175 (50.3)	204 (58.6)	128 (36.8)	

## Abbreviations

The following abbreviations are used in this manuscript:

EBV	Epstein-Barr virus
NMGDs	non-malignant gastroduodenal disorders
PUD	Peptic ulcer disease
GERD	gastroesophageal reflux disease
NSAIDS	nonsteroidal anti-inflammatory drugs
FD	functional dyspepsia
BabA	blood group antigen binding adhesion
OipA	outer inflammatory protein A
SabA	sialic acid-binding adhesion
VacA	vacuolating cytotoxin A
CagA	cytotoxin associated gene A
CTL	cytotoxic T lymphocytes
NK	natural killer cells
TNF-α	Tumor necrosis factor-α
IFN-γ	interferon-γ
TLR9	toll-like receptor 9
MMP-1	matrix metalloprotease-1
PRISMA	Preferred Reporting Items for Systematic Reviews and Meta-Analyses
EBER	EBV-encoded RNA
EBNA	Epstein–Barr nuclear antigens
VCA	viral capsid antigen
EA	early antigen
PMNC	Polymorphonuclear cells
MNC	mononuclear cells
NAG	Non-atrophic gastritis
BZLF1	BamHI Z Leftward reading Frame 1
BARF1	means BamHI A rightward open-reading frame-1
HCMV	human cytomegalovirus
